# C-954-10. Increasing Burn Admissions Include More Homelessness: A Single Centre Study from 2018-2024

**DOI:** 10.1093/jbcr/irag033.142

**Published:** 2026-04-06

**Authors:** Rachel Kidd, Lujia Long, Johanna Atienza-Serrano, Hua Shen, Vincent Gabriel, Aniela dela Cruz

**Affiliations:** University of Calgary, Calgary, AB; University of Calgary, Calgary, AB; Alberta Health Services, Calgary, AB; University of Calgary, Calgary, AB; University of Calgary, Calgary, AB; University of Calgary, Calgary, AB

## Abstract

**Introduction:**

Burn care requires a complete understanding of the patient’s physical and social circumstances. Clinical observation in one urban burn centre indicated an increasing socioeconomic disparity among patients. The purpose of this study was to analyze burn registry data to define perceived change in demographic trends in a single burn centre, identify differences in outcomes, particularly in relation to social determinants of health to inform local burn care.

**Methods:**

This study was approved by the institutional health ethics board. Data was extracted from the institutional NTRACS burn registry based on the ABA Burn Care Quality Platform data dictionary for burn patients aged 14-89 from January 1, 2018 to December 31, 2024. R was used for analysis. Descriptive statistics were used to demonstrate burn patient characteristics. Negative binomial models were used to assess burn trends with grouped logistic regression modeling used to assess the proportion of people experiencing homelessness (PEH) burns over time.

Logistic binary regressions were performed on cases with complete data to assess risk factors associated with outcomes. The independent variables for analysis included gender, age, marital status, living situation, inhalation injury, temperature, GCS, ETOH use, comorbidities, complications, TBSA and number of surgical procedures. The outcome variables for analysis included ICU admission, ventilator support, length of stay and discharge status.

**Results:**

We identified 749 burn patients during the study period, of which 131 were PEH. Overall burn injuries and PEH burns increased during this period, with the odds of a burn experiencing homelessness rising 42% per year over year. PEH were significantly more likely to live alone. 217 patients were included in our logistic regressions. We did not find evidence of an association between PEH and ICU admission, ventilator use, length of stay or discharge status. The total number of PEH in our sample may have limited statistical power to detect associations, though we cannot rule out other explanations such as a true lack of effect or residual confounding. ETOH use was a significant factor in several outcomes, with fourfold increase in risk of ICU admission and 13x increased risk of ventilator use. Age-related results included every additional year of age increasing both odds of mortality and 3% increase in odds of prolonged hospital stay. Limitations include the single centre setting and the presence of missing data, which reduced the sample size available for inferential analysis.

**Conclusions:**

The results of the study enhance understanding of local burn demographics. Further investigation is required to understand if the increase in PEH is proportionate to the local catchment area.

**Applicability of Research to Practice:**

The results of this study will be shared with stakeholders, including burn centre staff and those working with PEH in the local area. It will inform thesis work on vulnerable populations with burn injury.

**Funding for the study:**

Unrestricted Foundation/Philanthropy Grant.

